# Predictive Value of On-Treatment Alpha-Fetoprotein Change for Lenvatinib Response in Patients with Hepatocellular Carcinoma: A Real-World Retrospective Study in Vietnam

**DOI:** 10.3390/medicina62091774

**Published:** 2026-09-16

**Authors:** Ngoc-Tan Hoang, Van-Quang Le, Thang Tran, Thi-Que Pham, Thi-Dung Nguyen, Thi-Cuc Hoang, Thanh-Phuong Pham, Thi-Hoa Nguyen

**Affiliations:** 1 Department of Oncology, Hanoi Medical University, No. 1 Ton That Tung Street, Hanoi 100000, Vietnam; 2 Vietnam National Cancer Hospital, No. 30 Cau Buou Street, Thanh Liet Ward, Hanoi 100000, Vietnam; 3 Hanoi Oncology Hospital, No. 42A Thanh Nhan Street, Hanoi 100000, Vietnam

**Keywords:** alpha-fetoprotein, hepatocellular carcinoma, lenvatinib, mRECIST, treatment response, real-world data

## Abstract

*Background and Objectives*: Changes in alpha-fetoprotein (AFP) after initiating lenvatinib may reflect response in patients with hepatocellular carcinoma (HCC), but the optimal assessment time point and AFP decline threshold remain unstandardized. This study evaluated the association between AFP change at the first post-treatment measurement and imaging response according to the modified Response Evaluation Criteria in Solid Tumors (mRECIST). *Materials and Methods*: This retrospective study included 191 patients with HCC treated with lenvatinib. The primary analysis population comprised patients with pretreatment AFP ≥ 20 ng/mL and ≥1 post-treatment AFP measurement. AFP response was defined as a ≥40% decrease from pretreatment levels. The primary endpoints were the objective response rate (ORR) and disease control rate (DCR) according to mRECIST; progression-free survival (PFS) and overall survival (OS) were exploratory. Associations were assessed using multivariable logistic regression adjusted for AFP timing, albumin–bilirubin (ALBI) score, macrovascular invasion, and tumor number ≥ 5. Sensitivity and 2-month landmark analyses were also performed. *Results*: Of 124 patients, 47 (37.9%) achieved an AFP response. ORR was 44.7% versus 6.5% in the non-response group (OR 11.36; 95% confidence interval [CI] 3.68–42.67; *p* < 0.001); DCR was 83.0% versus 57.1% (OR 3.62; 95% CI 1.42–10.19; *p* = 0.003). After adjustment, AFP response remained independently associated with ORR (aOR 12.96; 95% CI 3.85–43.66; *p* < 0.001) and DCR (aOR 5.76; 95% CI 1.95–17.00; *p* = 0.002); findings were unchanged when restricted to cycle-1 measurements (aOR 12.27). In exploratory analyses, median PFS was 8.51 versus 4.30 months and median OS 18.53 versus 10.15 months; landmark analysis gave concordant estimates (PFS HR 0.38; OS HR 0.45). *Conclusions*: A ≥40% AFP decline at the first post-treatment measurement was independently associated with ORR and DCR according to mRECIST in patients with HCC and pretreatment AFP ≥ 20 ng/mL. These findings are hypothesis-generating and require confirmation in prospective, multicenter studies with independent validation before AFP kinetics can be considered in routine practice.

## 1. Introduction

Hepatocellular carcinoma (HCC) is the most common form of primary liver cancer and remains among the leading causes of cancer-related death worldwide, with an estimated 843,000 new cases and 732,000 deaths in 2024 [1]. Current clinical practice guidelines, based on the Barcelona Clinic Liver Cancer (BCLC) staging system, guide systemic treatment selection for patients with advanced HCC or those no longer eligible for locoregional therapy [2,3]. Atezolizumab plus bevacizumab is now the preferred first-line systemic option in many guidelines, based on the phase III IMbrave150 trial [4], while the STRIDE regimen (a single priming dose of tremelimumab plus regular-interval durvalumab) from the phase III HIMALAYA trial is another approved first-line systemic option [5]. Nevertheless, lenvatinib remains an important option for patients unsuitable for immunotherapy or in settings with limited access to immune checkpoint inhibitors (ICIs). Lenvatinib demonstrated non-inferiority to sorafenib for overall survival and achieved a higher objective response rate in the phase III REFLECT trial, establishing it as a standard systemic treatment for unresectable HCC [6]. In clinical practice, response to lenvatinib is currently assessed mainly by imaging according to the modified Response Evaluation Criteria in Solid Tumors (mRECIST), with the first assessment typically performed approximately 8 weeks after treatment initiation. This interval can create a lag between the onset of the biological response and its confirmation by imaging. By contrast, alpha-fetoprotein (AFP) is a routine, low-cost biomarker that can be measured repeatedly and early during treatment. Changes in AFP concentration after treatment initiation may therefore provide an early signal of tumor responsiveness before response is confirmed by imaging. Determining whether early AFP change after starting lenvatinib is associated with mRECIST response could provide useful additional information for treatment assessment and monitoring in clinical practice.

Prior studies on lenvatinib have shown that AFP change can emerge as early as weeks 2–4 and is associated with imaging response [7,8]. Saeki et al. defined AFP response as a ≥40% decrease after one month in patients with pretreatment AFP ≥ 10 ng/mL and reported a strong association with mRECIST response [8]. Liu et al. used a pretreatment AFP threshold of ≥20 ng/mL and a >20% decrease after four weeks, showing that the AFP-response group had better ORR, DCR, and PFS [9]. A meta-analysis of 26 studies comprising 3056 patients also showed that AFP decline was associated with more favorable OS and PFS, while noting substantial heterogeneity in cutoffs and assessment timing [10]. Recently, a multicenter study of 553 patients treated with lenvatinib showed that AFP trajectories over time could stratify OS and PFS [11], while a post hoc analysis of the REFLECT trial using a ≥20% AFP decline threshold at week 8 reported higher ORR, PFS, and OS in the response group [12]. Nevertheless, existing studies remain inconsistent regarding the optimal assessment time point and decline threshold, and they have not established AFP as a surrogate for imaging response or survival outcomes.

In this context, we used the first available post-treatment AFP result after lenvatinib initiation to evaluate the association between AFP change and imaging response according to mRECIST under real-world clinical practice conditions. The primary analysis was performed in patients with pretreatment AFP ≥ 20 ng/mL, with AFP response defined as a ≥40% decrease from the pretreatment value. Alternative AFP decline thresholds were used in sensitivity analyses to assess the robustness of the findings.

## 2. Materials and Methods

### 2.1. Study Design and Population

This retrospective study included 191 patients with HCC treated with lenvatinib in routine clinical practice at Vietnam National Cancer Hospital and Hanoi Oncology Hospital between March 2021 and December 2025 (data lock date: 1 June 2026). HCC diagnosis was established based on standard imaging and/or histopathological criteria. All 191 patients (the overall population) were included to describe the general characteristics of the study. No formal sample size calculation was performed; the study included all consecutive eligible patients treated during the study period, consistent with the retrospective design.

**Figure 1:**
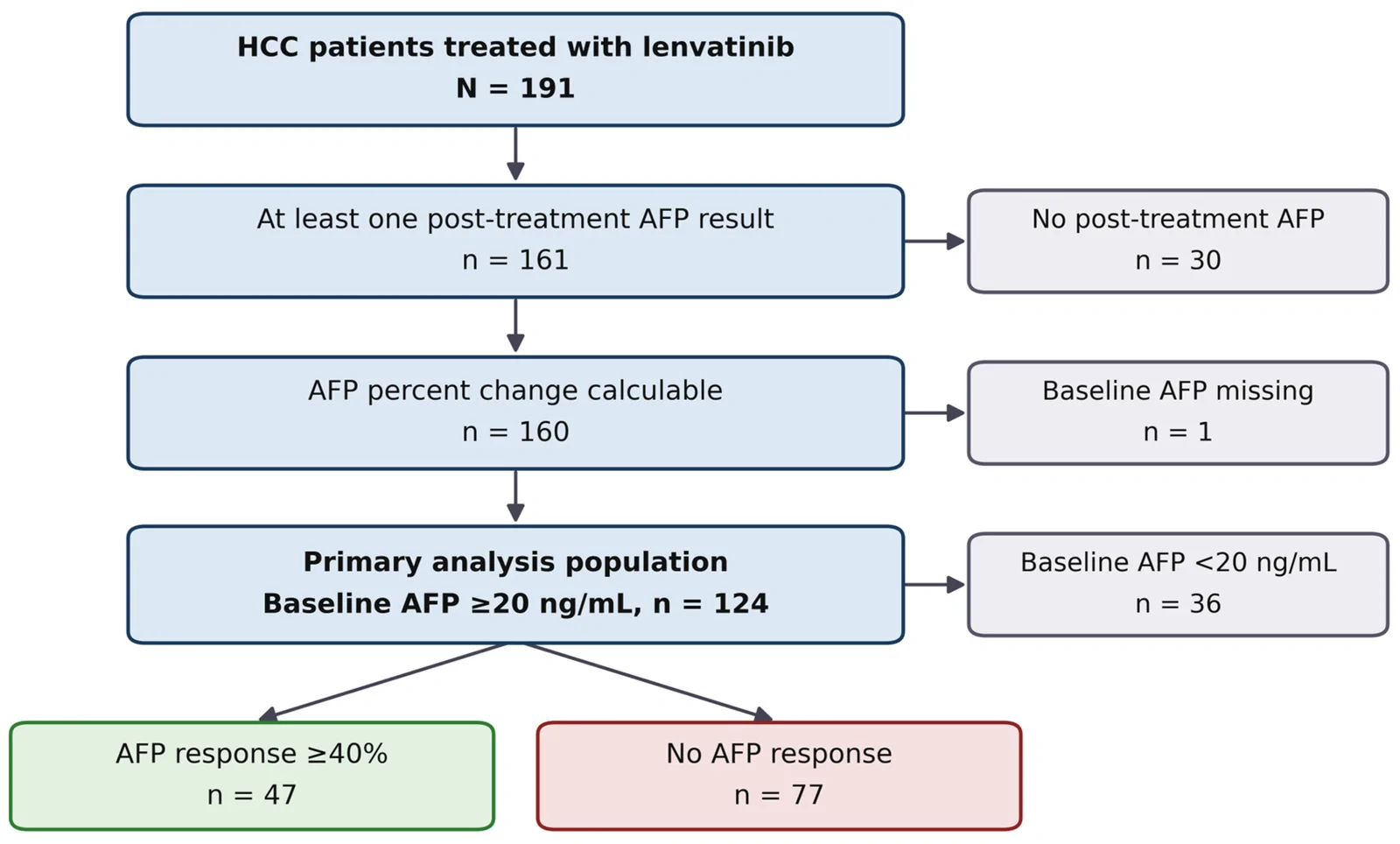
Flow diagram of the analysis population selection. Of the 191 patients treated with lenvatinib, 124 patients with pretreatment AFP ≥ 20 ng/mL and a valid post-treatment AFP were included in the primary analysis.

The study population was stratified according to two independent criteria to serve different analyses, illustrated in detail in the patient selection flow diagram (Figure 1). The first criterion was pretreatment AFP availability: one patient without a pretreatment AFP value was considered to have missing data and was excluded from all AFP-related analyses; the remaining 190 patients had a valid pretreatment AFP value, comprising 47 patients with AFP < 20 ng/mL and 143 patients with AFP ≥ 20 ng/mL. This population of 190 patients with valid pretreatment AFP was used for the supplementary analysis comparing ORR, DCR, PFS, and OS according to the pretreatment AFP threshold (Section 2.4), regardless of post-treatment AFP availability. The second criterion was the availability of post-treatment AFP: 161 patients (84.3%) had at least one AFP measurement after treatment initiation, allowing calculation of the AFP change rate; the remaining 30 patients without post-treatment AFP are shown in the selection flow diagram and were descriptively compared with the 161 patients who had post-treatment AFP to assess the potential for selection bias. This comparison was made both in the whole cohort and, in a second panel, among patients with pretreatment AFP ≥ 20 ng/mL who would otherwise have been eligible for the primary analysis (Table S1).

The primary analysis population—used for the primary endpoints regarding AFP response (Section 2.4)—is the intersection of the two criteria above: patients with both pretreatment AFP ≥ 20 ng/mL and at least one post-treatment AFP measurement, comprising 124 patients. The supplementary analysis by pretreatment AFP (comparing the <20 ng/mL and ≥20 ng/mL groups) was performed in all 190 patients with a valid pretreatment AFP value, as described above, regardless of post-treatment AFP testing.

### 2.2. Treatment and Assessment

The starting dose of lenvatinib (Lenvima, Eisai Co., Ltd., Tokyo, Japan) was weight-based: 12 mg orally once daily in patients ≥ 60 kg and 8 mg orally once daily in patients < 60 kg. Lenvatinib was administered continuously without a scheduled rest period; for the purpose of laboratory follow-up, one treatment cycle was defined as 30 days (one month). The actual dose could be adjusted by the treating physician; dose reduction or temporary interruption was implemented based on toxicity and the patient’s clinical status. Pretreatment characteristics included age, sex, Eastern Cooperative Oncology Group (ECOG) performance status, Child–Pugh score, albumin–bilirubin (ALBI) grade [13], BCLC stage, macrovascular invasion (including main portal trunk thrombus, VP4), extrahepatic metastasis, tumor size, tumor number, hepatitis B virus (HBV) and hepatitis C virus (HCV) status, diabetes mellitus, alcohol use, prior local treatment (surgery, transarterial chemoembolization [TACE], or radiofrequency ablation [RFA]), and pretreatment AFP. Lenvatinib treatment duration and the reason for treatment discontinuation, when applicable, were also recorded.

The first imaging assessment was typically performed approximately 8 weeks after treatment initiation—that is, after approximately two treatment cycles—and thereafter according to routine practice at each center, approximately every 8 weeks. Among patients who achieved an objective response, the median time to documented response was 2.0 months. Tumor response was assessed according to mRECIST [14] and classified as complete response (CR), partial response (PR), stable disease (SD), or progressive disease (PD). The objective response rate (ORR) was defined as CR + PR, and the disease control rate (DCR) was defined as CR + PR + SD.

Serum AFP was measured by electrochemiluminescence immunoassay (ECLIA) using the Elecsys AFP assay on cobas e 402/e 801 analyzers (Roche Diagnostics, Mannheim, Germany), according to the manufacturer’s instructions. The assay was standardized against the 1st WHO International Reference Preparation 72/225 and had a measuring range of 0.908–1210 ng/mL; samples exceeding the upper limit were reanalyzed after 1:50 dilution. The limit of quantitation was 2.72 ng/mL, and intermediate precision (coefficient of variation) ranged from 2.3% to 4.2% across serum concentrations of 2.18–554 ng/mL. In each patient, pretreatment and post-treatment samples were assayed by the same method on the same platform, in the clinical laboratory of the treating hospital under routine internal quality control.

For AFP analyses, the first post-treatment AFP result was used to calculate the percentage change from the pretreatment value.

### 2.3. Definition of First Post-Treatment AFP and AFP Response

The first post-treatment AFP was defined as the first valid AFP result recorded after lenvatinib initiation. No fixed assessment time point was imposed; the treatment cycle at which the first AFP was recorded was captured and entered into the adjusted analyses. Because one cycle corresponded to 30 days, a first AFP recorded at cycle 1, 2, 3, or 4 would correspond to approximately 1, 2, 3, or 4 months after lenvatinib initiation, respectively.

The percentage change in AFP was calculated as

AFP change (%) = 100 × (first post-treatment AFP − pretreatment AFP)/pretreatment AFP.

The primary AFP response was defined as a decrease of ≥40%, equivalent to a post-treatment/pretreatment AFP ratio ≤ 0.60. The 40% threshold was pre-specified based on existing literature (not derived from this study’s own dataset). Alternative AFP decline thresholds of ≥20%, ≥30%, and ≥50% were used to assess the robustness of the primary findings.

### 2.4. Endpoints

The primary endpoints were the associations between AFP response (≥40%) and both ORR and DCR according to mRECIST. Secondary survival endpoints included PFS and OS. The discriminative ability of continuous AFP change for imaging response, as well as sensitivity, specificity, positive predictive value, negative predictive value, alternative AFP decline thresholds, and concordance between AFP and mRECIST, was explored in supplementary analyses. A separate supplementary analysis evaluated ORR, DCR, PFS, and OS according to two pretreatment AFP groups (<20 and ≥20 ng/mL).

PFS was calculated from the date of lenvatinib initiation to progression or death; OS was calculated to death from any cause. Although PFS and OS are secondary survival endpoints, AFP response is a post-treatment variable measured at non-uniform time points; therefore, the survival analyses were considered exploratory and subject to the risk of immortal-time (guarantee-time bias [15]; a landmark analysis was therefore performed to address this risk (Section 2.5).

### 2.5. Statistical Analysis

Continuous variables were described using medians and interquartile ranges; categorical variables were described using frequencies and percentages. Fisher’s exact test was used for the crude association between AFP response and ORR/DCR. For the primary endpoints, binary logistic regression models for ORR and DCR included AFP response (≥40% decline) as the exposure of interest, with four covariates specified a priori on clinical grounds (not through stepwise or other data-driven selection): timing of the first AFP measurement (after cycle 1 vs. cycle 1, binary), pretreatment ALBI score (continuous), macrovascular invasion (binary), and tumor number ≥ 5 (binary). Models were fitted using complete-case analysis; patients with a missing value for any covariate were excluded from the corresponding model. The number of covariates was limited to be commensurate with the number of response events. Median follow-up was estimated using the reverse Kaplan–Meier method.

Receiver operating characteristic (ROC) curves, summarized by the area under the curve (AUC), were used to assess the discriminative ability of continuous AFP change. The optimal cutoff determined by the Youden index is presented only as an exploratory analysis and does not replace the pre-specified ≥40% AFP decline threshold. For the secondary endpoints PFS and OS, multivariable Cox models used the same set of adjustment covariates; because the exposure variable was defined after treatment initiation, these results were considered exploratory and interpreted cautiously owing to the risk of immortal-time bias. With 5 adjustment covariates and a complete-case population of 113 patients, the events-per-variable (EPV) ratio was approximately 19.4 for PFS (97 events) and 16.4 for OS (82 events), above the commonly recommended minimum of 10 events per variable, limiting the risk of overfitting. In the supplementary analysis by pretreatment AFP, ORR and DCR were compared using Fisher’s exact test, while PFS and OS were estimated using the Kaplan–Meier method and compared using the log-rank test. Adjusted models included pretreatment ALBI score, macrovascular invasion, and tumor number ≥ 5. Because these are supplementary analyses without adjustment for multiple testing, *p*-values were interpreted cautiously. All tests were two-sided, and *p* < 0.05 was considered statistically significant. Analyses were performed using R (v4.6.0; R Foundation for Statistical Computing, Vienna, Austria). The sensitivity and landmark analyses were performed in Python (v3.10.12) using the lifelines (v0.30.0) and statsmodels (v0.14.6) packages. The study was reported according to the Strengthening the Reporting of Observational Studies in Epidemiology (STROBE) guideline [16] for observational studies (the STROBE checklist is provided in the Supplementary Materials, Table S2).

Two additional analyses were performed to address the non-uniform timing of AFP assessment and the risk of immortal-time bias. First, a sensitivity analysis repeated the ORR, DCR, and survival analyses restricted to patients whose first post-treatment AFP was obtained at cycle 1, thereby fixing the assessment time point at approximately one month. Second, a landmark analysis was performed with the landmark set at 2 months after lenvatinib initiation, corresponding to the end of cycle 2, by which time AFP response status had been determined in 118 of 124 patients (95.2%). Patients who died or progressed before the landmark were excluded from the respective analysis, and survival time was recomputed from the landmark; a landmark at 3 months was examined as a further check. Both analyses used the same adjustment covariates as the primary models.

### 2.6. Research Ethics

This study used retrospective medical record data, maintained patient confidentiality, and did not interfere with the treatment process. The study was approved by the Institutional Review Board for Biomedical Research, Hanoi Medical University (approval no. 935/GCN-HĐĐĐNCYSH-ĐHYHN, date of approval 16 June 2023; reference code IRB-VN01.001/IRB00003121/FWA00004148). The study was conducted in accordance with the principles of the Declaration of Helsinki.

## 3. Results

### 3.1. Analysis Population

Table 1 summarizes the baseline characteristics of the overall study population (N = 191). The median follow-up, estimated using the reverse Kaplan–Meier method, was 24.8 months (95% CI 20.2–27.3).

Among 191 patients, 161 (84.3%) had at least one post-treatment AFP measurement and 30 (15.7%) did not. One patient had no pretreatment AFP value (considered missing), and the remaining 190 patients had a valid pretreatment AFP, comprising 47 patients with AFP < 20 ng/mL and 143 patients with AFP ≥ 20 ng/mL. Among patients with post-treatment AFP, 160 had a calculable percentage AFP change, comprising 36 patients with pretreatment AFP < 20 ng/mL and 124 patients with pretreatment AFP ≥ 20 ng/mL. This group of 124 patients constituted the primary AFP-kinetics analysis population.

The 30 patients without a post-treatment AFP measurement had no AFP result recorded at any of the 12 follow-up cycles, indicating that testing was not requested rather than performed outside the analysis window. Their treatment course was not truncated: the shortest lenvatinib duration in this group was 2.0 months (median 7.0; IQR 6.0–10.0), 27 of 30 (90.0%) were treated for at least 3 months, and none died within the first 3 months. The proportion without post-treatment AFP differed between the two participating hospitals (9/107, 8.4% vs. 21/84, 25.0%; *p* = 0.002), consistent with differing institutional monitoring practices rather than differing disease course. Among patients who would otherwise have been eligible, 19 of 143 (13.3%) with pretreatment AFP ≥ 20 ng/mL lacked a post-treatment AFP; compared with the 124 patients in the primary analysis population, these 19 patients had less advanced disease and better outcomes (BCLC stage C 57.9% vs. 79.8%, *p* = 0.044; death 36.8% vs. 67.7%, *p* = 0.019; progression or death 52.6% vs. 84.7%, *p* = 0.003; median PFS 12.29 vs. 5.95 months, *p* = 0.014; Table S1). Exclusion of these patients therefore shifts the primary analysis toward a more advanced population and is unlikely to have inflated the association between AFP response and favorable outcome.

In the primary analysis population, the first AFP result was recorded at cycle 1 (99 patients), cycle 2 (19 patients), cycle 3 (4 patients), and cycle 4 (2 patients) (Table 2). Therefore, if only cases with an AFP result at cycle 1 were accepted, 25/124 (20.2%) patients would be excluded from the analysis, substantially reducing the study’s sample size.

### 3.2. AFP Response and mRECIST Response

Forty-seven of 124 patients (37.9%) had an AFP decline ≥40%. ORR was 44.7% (21/47) in the AFP-response group, compared with 6.5% (5/77) in the non-response group. AFP response was associated with approximately 11-fold higher odds of imaging response (odds ratio [OR], 11.36; 95% confidence interval [CI], 3.68–42.67; *p* < 0.001).

**Figure 2:**
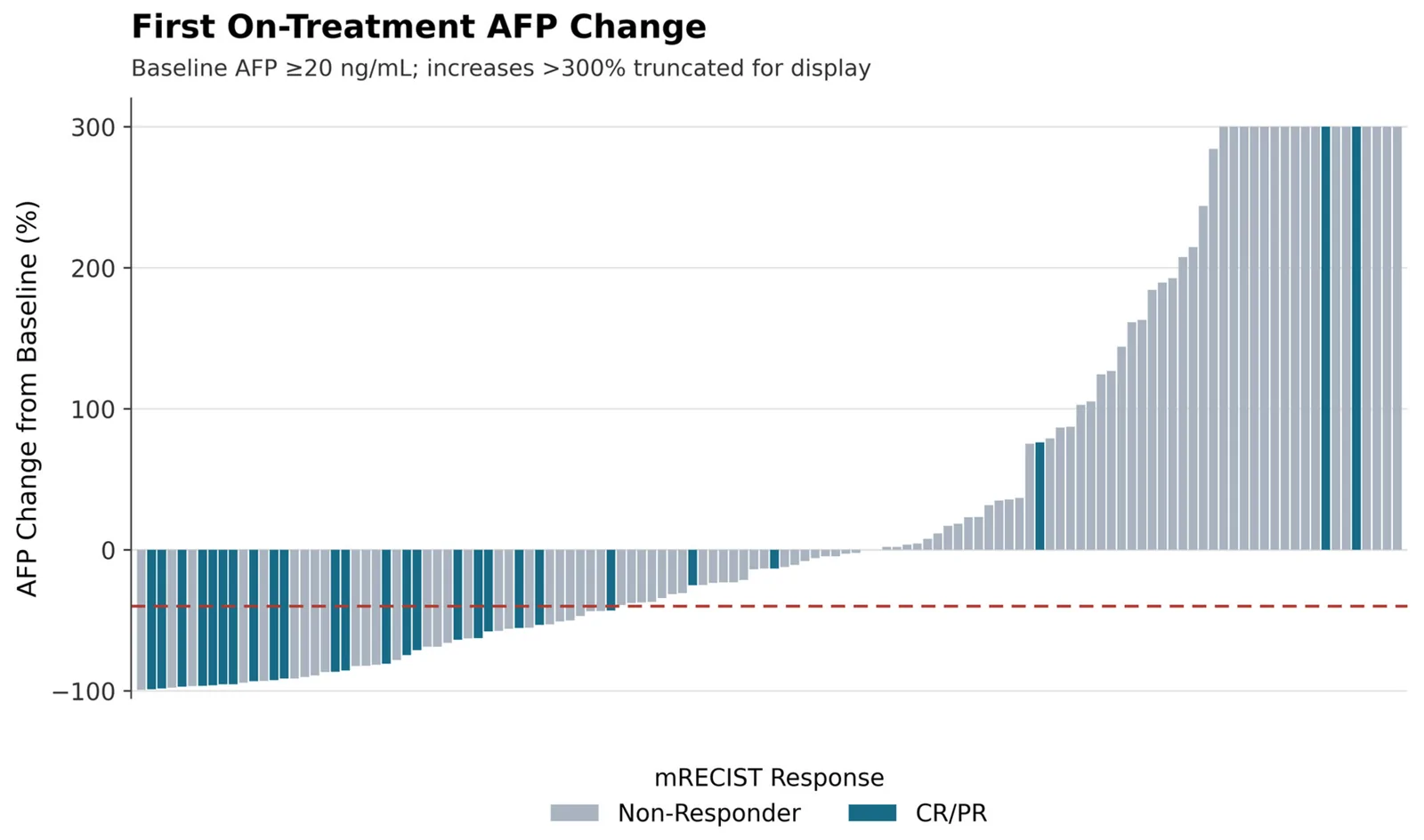
First post-treatment AFP change. Waterfall plot of 124 patients with pretreatment AFP ≥ 20 ng/mL. The dashed line indicates the 40% decline threshold. Colors indicate mRECIST response; AFP increases >300% were truncated for display purposes.

DCR was 83.0% (39/47) in the AFP-response group and 57.1% (44/77) in the non-response group (OR 3.62; 95% CI 1.42–10.19; *p* = 0.003). Individual patient-level AFP changes are shown in the waterfall plot (Figure 2). Crude associations are summarized in Table 3.

### 3.3. Multivariable Analysis

Multivariable logistic regression was used to test whether the association between AFP response and ORR/DCR observed in the crude analysis (Table 3) remained statistically significant after adjustment for four covariates specified a priori on clinical grounds (Section 2.5): timing of the first AFP measurement, pretreatment ALBI score, macrovascular invasion, and tumor number ≥ 5.

Of the 124 patients in the primary analysis population, 11 (8.9%) had a missing value for at least one adjustment covariate (5 missing ALBI scores, 6 missing tumor numbers) and were excluded from the complete-case multivariable model, leaving 113 patients. In this model, AFP decline ≥40% remained independently associated with ORR (adjusted OR 12.96; 95% CI 3.85–43.66; *p* < 0.001). AFP response was also independently associated with DCR (adjusted OR 5.76; 95% CI 1.95–17.00; *p* = 0.002) (Table 4).

The timing of the first AFP measurement (after cycle 1 versus earlier) was not independently associated with ORR (aOR 1.75; *p* = 0.394).

### 3.4. Analysis of AFP Decline Thresholds and Predictive Value

An association between AFP decline and ORR was observed at all four thresholds tested—20%, 30%, 40%, and 50% (Table 5)—supporting the robustness of the AFP–response relationship across a range of cutoffs. The 40% threshold was retained for the primary analysis because it was pre-specified from prior lenvatinib literature [8,17], not because it was empirically optimal in this dataset. A 40% decline is also large relative to measurement error: the intermediate precision of the AFP assay used here was 2.3–4.2%, so a 40% change is approximately an order of magnitude greater than the analytical variability and is unlikely to reflect technical noise. Smaller thresholds such as 20% are correspondingly more susceptible to biological fluctuation unrelated to treatment. The 40% threshold further lies mid-range among the 20–50% thresholds reported in the literature, offering a reasonable balance between sensitivity and specificity. The AUC for continuous AFP change was 0.794 (95% CI 0.685–0.903; Figure S2). For comparison only, the data-derived optimal cutoff by the Youden index was approximately 53% (sensitivity 76.9%, specificity 79.6%); because this cutoff was derived post hoc from the study’s own data, it carries a risk of overfitting and is reported as an exploratory benchmark rather than a recommended threshold.

### 3.5. Concordance Between AFP Response and Imaging Response

Patients were classified into four groups according to AFP response (≥40% decline) and imaging response by mRECIST (CR/PR). Twenty-one patients responded by both AFP and mRECIST, 72 patients responded by neither criterion, 26 patients had an AFP response without achieving CR/PR, and 5 patients achieved CR/PR without an AFP response.

Median PFS and OS in the group responding by both AFP and mRECIST were 17.25 months and not reached, respectively, compared with 4.07 and 9.53 months in the group responding by neither criterion. In the two groups with discordant AFP and mRECIST results, PFS and OS tracked more closely with mRECIST response than with AFP response: the AFP−/mRECIST+ group (n = 5) had a median PFS of 15.21 months and OS of 15.74 months—comparable to the group responding by both criteria—whereas the AFP+/mRECIST− group had a median PFS of 5.32 months and OS of 11.73 months, closer to the group responding by neither criterion. Because the AFP−/mRECIST+ group was very small (n = 5), these estimates have wide uncertainty and should be interpreted with caution. Because both AFP response and imaging response are determined after treatment initiation, this analysis is descriptive only, is intended to illustrate prognostic stratification, and should not be interpreted as evidence of a causal relationship (Table 6).

**Table 6:** Stratification by AFP response and mRECIST response.

Phenotype	n	DCR	Median PFS, Months	Median OS, Months
AFP+/mRECIST+	21	100%	17.25	Not reached
AFP+/mRECIST−	26	69.2%	5.32	11.73
AFP−/mRECIST+	5	100%	15.21	15.74
AFP−/mRECIST−	72	54.2%	4.07	9.53

**Figure 3:**
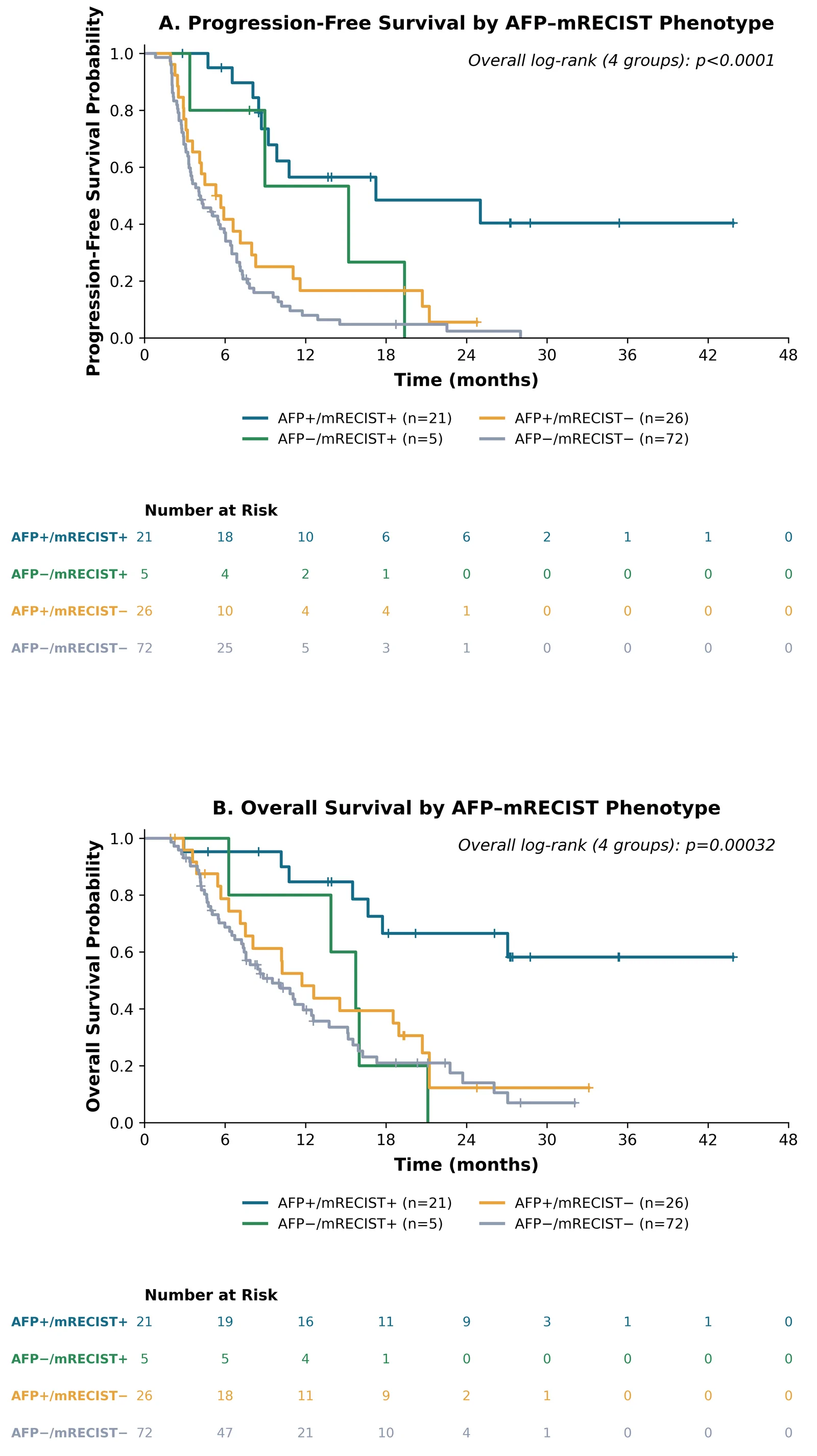
Survival by AFP–mRECIST phenotype (Table 6). Kaplan–Meier curves for progression-free survival (**A**) and overall survival (**B**), with number-at-risk tables at 6-month intervals, across the four AFP–mRECIST concordance groups: AFP+/mRECIST+, AFP−/mRECIST+, AFP+/mRECIST−, and AFP−/mRECIST−. Overall (4-group) log-rank test: PFS *p* < 0.0001 (χ^2^ = 31.48, df = 3); OS *p* = 0.00032 (χ^2^ = 18.66, df = 3). The AFP−/mRECIST+ group was small (n = 5), so estimates for this group should be interpreted with caution.

DCR: tumor response by mRECIST (CR + PR + SD); PFS and OS: Kaplan–Meier estimates. Overall (4-group) log-rank test (df = 3): PFS *p* < 0.0001 (χ^2^ = 31.48); OS *p* = 0.00032 (χ^2^ = 18.66). The corresponding Kaplan–Meier curves and number-at-risk table are presented in Figure 3.

### 3.6. Survival Analysis

**Figure 4:**
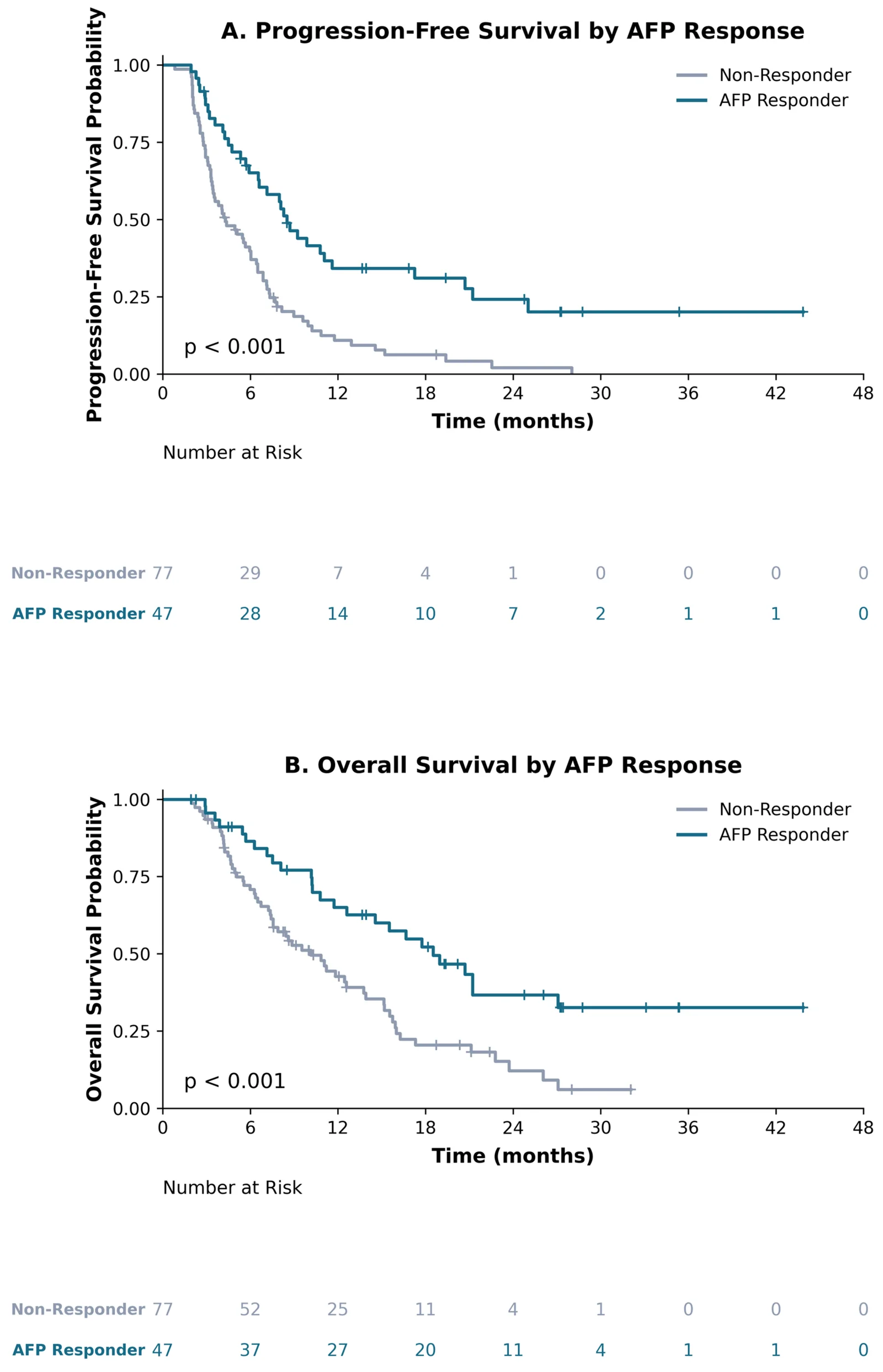
Survival according to AFP response. Kaplan–Meier curves for PFS (**A**) and OS (**B**), stratified by AFP decline ≥40% at the first post-treatment measurement, with a number-at-risk table at 6-month intervals. *P*-values are from the log-rank test (PFS: *p* < 0.0001; OS: *p* = 0.00076). This analysis is exploratory because AFP response is a post-treatment variable.

By Kaplan–Meier analysis, the AFP-response group had a median PFS of 8.51 months (95% CI 6.60–17.25), longer than that in the non-response group (4.30 months; 95% CI 3.42–6.04; log-rank *p* < 0.001). The corresponding median OS values were 18.53 months (95% CI 12.61–not reached) and 10.15 months (95% CI 7.56–13.90; log-rank *p* = 0.001) (Figure 4).

In the complete-case multivariable Cox model, among 113 patients with sufficient data for adjustment (97 progression/death events for PFS; 82 death events for OS), AFP response was associated with a lower risk of progression or death (hazard ratio [HR] 0.38; 95% CI 0.24–0.60; *p* < 0.001) and a lower risk of death (HR 0.49; 95% CI 0.30–0.79; *p* = 0.004). Tumor number ≥ 5 was also independently associated with worse PFS (HR 1.74; 95% CI 1.13–2.68; *p* = 0.012) and OS (HR 1.61; 95% CI 1.03–2.54; *p* = 0.039). Because AFP response is determined after treatment initiation, these estimates were re-examined using a landmark analysis (Section 3.10).

### 3.7. Supplementary Analysis by Pretreatment AFP

Among 190 patients with a valid pretreatment AFP, 47 had AFP < 20 ng/mL, and 143 had AFP ≥ 20 ng/mL. ORR was 36.2% and 21.7%, respectively (*p* = 0.055). DCR was higher in the AFP < 20 ng/mL group (87.2% versus 69.2%; *p* = 0.021). By Kaplan–Meier analysis, median PFS was 10.58 months in the AFP < 20 ng/mL group versus 6.50 months in the AFP ≥ 20 ng/mL group (log-rank *p* = 0.011); median OS was 22.37 and 12.61 months, respectively (log-rank *p* = 0.017) (Table 7).

In adjusted models among 173 patients with sufficient data, pretreatment AFP ≥ 20 ng/mL was associated with a borderline significantly lower ORR (OR 0.45; 95% CI 0.20–1.00; *p* = 0.050) and a significantly lower DCR (OR 0.34; 95% CI 0.12–0.95; *p* = 0.040), but was not significantly associated with PFS (HR 1.44; 95% CI 0.93–2.23; *p* = 0.102). Pretreatment AFP ≥ 20 ng/mL remained associated with a higher risk of death (HR 1.64; 95% CI 1.01–2.66; *p* = 0.046). Because this is a supplementary analysis, these results should be considered exploratory.

### 3.8. Subgroup Analysis by HBV Status

In the primary analysis population (n = 124), 60 patients (48.4%) were HBV-positive and 64 (51.6%) were HBV-negative; only 4 patients (3.2%) were HCV-positive, which was too few for a separate subgroup analysis. ORR, DCR, median PFS, and median OS did not differ significantly between the HBV+ and HBV− groups (*p* = 1.000, 1.000, 0.539, and 0.385, respectively), indicating that the two subgroups had broadly similar outcome profiles before stratification by AFP response.

The association between AFP response ≥40% and ORR, DCR, PFS, and OS was directionally consistent in both the HBV+ and HBV− subgroups (Table 8). The OR for ORR according to AFP response was 13.67 (95% CI 2.50–143.40) in the HBV+ subgroup and 9.34 (95% CI 2.01–60.95) in the HBV− subgroup; the corresponding HRs for PFS were 0.48 (95% CI 0.27–0.88) and 0.36 (95% CI 0.20–0.67). These findings suggest that the association between AFP response and treatment outcome does not depend substantially on HBV status. This is an exploratory subgroup analysis, unadjusted for liver-function and tumor-burden covariates and not corrected for multiple testing given the limited sample size of each subgroup; the results require confirmation in a larger cohort.

### 3.9. Sensitivity Analysis Restricted to Cycle-1 AFP Measurements

Because the timing of the first post-treatment AFP was not uniform, the primary analyses were repeated in the 99 patients whose first AFP was obtained at cycle 1, fixing the assessment time point at approximately one month. Thirty-five of these patients (35.4%) achieved an AFP response. ORR was 37.1% (13/35) versus 6.2% (4/64) (OR 8.64; 95% CI 2.35–40.32; *p* < 0.001), and DCR was 80.0% (28/35) versus 59.4% (38/64) (OR 2.71; 95% CI 0.97–8.48; *p* = 0.046). After adjustment for pretreatment ALBI score, macrovascular invasion, and tumor number ≥ 5, AFP response remained independently associated with ORR (aOR 12.27; 95% CI 3.01–50.09; *p* < 0.001) and with DCR (aOR 4.48; 95% CI 1.35–14.85; *p* = 0.014). Median PFS was 8.71 versus 4.30 months, and median OS was 18.96 versus 10.15 months (log-rank *p* < 0.001 and *p* = 0.002). These estimates closely match those of the primary analysis (Table 9).

### 3.10. Landmark Analysis

To address the risk of immortal-time bias, survival was re-analyzed from a landmark set at 2 months after lenvatinib initiation, corresponding to the end of cycle 2, by which time AFP response status had been determined in 118 of 124 patients (95.2%). Two patients had died, and four had progressed or died before the landmark and were excluded from the respective analyses. Measured from the landmark, AFP response remained associated with a lower risk of progression or death (HR 0.38; 95% CI 0.24–0.59; *p* < 0.001) and a lower risk of death (HR 0.45; 95% CI 0.28–0.73; *p* = 0.001); the corresponding adjusted estimates were HR 0.35 (95% CI 0.21–0.57) and HR 0.48 (95% CI 0.29–0.79). A landmark set at 3 months produced concordant estimates (Table 10; Figure S3). Because these hazard ratios are close to those obtained in the primary Cox models (PFS HR 0.38; OS HR 0.49), the observed association between early AFP response and survival is unlikely to be explained by immortal-time bias.

## 4. Discussion

This study showed that a ≥40% AFP decline at the first post-treatment measurement was strongly associated with imaging response according to mRECIST in patients with HCC treated with lenvatinib who had pretreatment AFP ≥ 20 ng/mL. The ORR of the AFP-response group was nearly seven-fold higher than that of the non-response group (44.7% versus 6.5%), and this association remained clear after adjustment for liver function, tumor burden, and assessment timing.

Our results are consistent with previous studies, while also extending the evidence to a real-world clinical practice setting. Kodama et al. reported that patients with a sustained AFP decline from week 2 to week 4 achieved an ORR of 67%, whereas no patients without a sustained AFP decline achieved an imaging response (0%; *p* = 0.02) [7]. Saeki et al. defined AFP response as a ≥40% decrease after 1 month in patients with pretreatment AFP ≥ 10 ng/mL and showed that the AFP-response group had markedly higher ORR (68.4% versus 7.1%) and DCR (84.2% versus 36.0%); AFP response was also an independent predictor of objective response (OR 51.39; 95% CI 4.89–540.28; *p* = 0.001) [8]. Similarly, Liu et al. used a >20% AFP decline threshold at 4 weeks in patients with HBV-related HCC and pretreatment AFP ≥ 20 ng/mL, reporting an ORR of 34.5% versus 6.3%, DCR of 82.8% versus 50.0%, and median PFS of 13 versus 7 months in the AFP-response and non-response groups [9]. In our study, ORR was 44.7% versus 6.5%, and DCR was 83.0% versus 57.1%, indicating a degree of clinical discrimination comparable to previous studies, despite a more flexible AFP assessment timing that reflects routine treatment practice.

Our survival findings are also consistent with existing evidence. A meta-analysis by Tian et al. of 3056 patients treated with targeted therapy or immunotherapy showed that AFP response was associated with a lower risk of death (pooled HR 0.48, 95% CI 0.40–0.56) and a lower risk of progression or death (pooled HR 0.39, 95% CI 0.33–0.46) [10]. A more recent meta-analysis by an overlapping author group, restricted to 131 studies of immune checkpoint inhibitor-treated patients, similarly found that AFP response was associated with more favorable OS (pooled HR 0.41, 95% CI 0.33–0.52), PFS (pooled HR 0.38, 95% CI 0.30–0.47), ORR (pooled OR 5.39, 95% CI 3.96–7.32), and DCR (pooled OR 5.48, 95% CI 3.71–8.11), and identified an AFP decline >20% as the most commonly used and efficient response threshold, while emphasizing that accurately defining early AFP response remains an area requiring further study [18]. In lenvatinib-treated patients, a 2025 multicenter study identified four AFP trajectory patterns and reported that the rapid-decline group had a lower risk of death (adjusted HR 0.28, 95% CI 0.18–0.42, *p* < 0.001) and a lower risk of progression or death (adjusted HR 0.34, 95% CI 0.24–0.47, *p* < 0.001) compared with the persistently high AFP group [11]. A 2026 post hoc analysis of the REFLECT trial also showed that patients with a ≥20% AFP decline at week 8 achieved a higher ORR by mRECIST (48.0% versus 13.7%), longer median PFS (7.4 versus 3.5 months), and longer median OS (13.4 versus 8.3 months) [12]. Similarly, in our study, AFP response was associated with longer PFS and OS (adjusted HR 0.38 and 0.49, respectively). A summary comparison with prior studies is presented in Table 11. However, cross-study comparisons should be interpreted cautiously given differences in study populations, AFP response criteria, assessment timing, treatment regimens, and analytical methods.

The novel aspects of this study, relative to previously published AFP–lenvatinib/AFP–ICI studies and meta-analyses (Table 11), are (1) the use of the first available post-treatment AFP result in clinical practice, rather than a fixed testing time point, which more closely reflects routine monitoring and limits patient exclusion; (2) the concordance analysis between AFP response and imaging response across four AFP–mRECIST phenotypes (Table 6), clarifying the complementary—not substitutive—role of AFP in treatment monitoring; and (3) the assessment of the consistency of the AFP response–outcome association according to HBV status (Table 8), an aspect not previously reported in AFP–lenvatinib studies.

The use of the first post-treatment AFP result reflects routine clinical practice but introduces heterogeneity in assessment timing. To mitigate this factor, we recorded the timing of the first AFP measurement, included this variable in the multivariable model, and repeated the primary analysis in the 99 patients who were measured at cycle 1. Timing was not independently associated with objective response (aOR 1.75; *p* = 0.394), and the cycle-1 analysis reproduced the primary estimate almost exactly (aOR 12.27 versus 12.96; Section 3.9). However, these measures cannot fully substitute for standardizing assessment timing in prospective studies. Accordingly, our findings should be interpreted as evidence supporting the utility of a flexible AFP monitoring strategy in clinical practice, rather than as an evaluation of AFP testing performance at one fixed post-treatment time point.

There is no universally accepted AFP threshold. A 20% threshold has been used in the Liu study, the meta-analysis, and the REFLECT analysis [9,10,12], while a 40% threshold was used at week 4 in the Saeki study and within 8 weeks in the Hsu study [8,17]. In this dataset, the association with ORR was consistent across all four thresholds of 20%, 30%, 40%, and 50% (Figure S1). The internally derived optimal cutoff of approximately 53% is exploratory only, as it was generated from this dataset and carries the risk of overfitting. Therefore, selecting 40% as the primary analysis threshold was a pre-specified decision grounded in the literature rather than an empirically optimized choice; results at the other thresholds should be viewed as sensitivity analyses rather than a search for a universal cutoff.

Analysis of discordant cases between AFP response and mRECIST response also yielded clinically meaningful information. Some patients had a marked AFP decline but had not yet achieved CR or PR by mRECIST, possibly because imaging assessment was performed early, the disease had been stable for a prolonged period, or there was a discrepancy between changes in tumor biological activity and imaging response. Conversely, some patients achieved an imaging response without a ≥40% AFP decline. These findings suggest that AFP should not be used alone to decide whether to continue or discontinue lenvatinib, but should be interpreted together with imaging response, clinical course, and liver function.

How an early AFP result might influence management before the first scheduled imaging assessment deserves explicit comment. In our view, an early AFP response should not by itself trigger continuation, modification, or discontinuation of therapy, and it does not substitute for imaging. Its role is to add biological information during the interval before the first scan, which in this cohort was approximately 8 weeks. In a patient with a marked AFP decline who is clinically stable and tolerating treatment, this result provides supportive evidence of biological activity while awaiting imaging. Conversely, the absence of an early AFP decline should not be read as treatment failure: AFP kinetics vary between patients, and five patients in this cohort achieved an imaging response without an early AFP decline. In such patients, the appropriate response is closer clinical monitoring and consideration of earlier imaging, rather than a change in therapy based on AFP alone. AFP kinetics should therefore be regarded as a supportive biomarker, and prospective studies are required to determine whether AFP-informed decisions improve clinical outcomes.

Analysis by pretreatment AFP adds a perspective distinct from post-treatment AFP kinetics. Patients with pretreatment AFP < 20 ng/mL had more favorable DCR, PFS, and OS in the unadjusted analysis. After adjustment, the association remained statistically significant for OS and DCR, was of borderline significance for ORR, and was no longer statistically significant for PFS. This finding suggests that a low pretreatment AFP may reflect a lower disease burden or more favorable biological characteristics, but is insufficient to establish pretreatment AFP as an independent prognostic factor for all outcomes. Conversely, assessment of AFP kinetics is only meaningful in patients with a sufficiently high pretreatment AFP, because in the pretreatment AFP < 20 ng/mL group, the relative AFP change is less stable and less likely to reflect the tumor’s biological response. Therefore, pretreatment AFP and post-treatment AFP response should be regarded as complementary rather than interchangeable pieces of information: pretreatment AFP reflects baseline disease characteristics, whereas AFP kinetics reflect the early biological response to treatment.

This study has several limitations. First, the timing of the first AFP measurement was not standardized, and AFP response is a variable determined after treatment initiation; the PFS and OS analyses are therefore susceptible to immortal-time bias. A sensitivity analysis restricted to cycle-1 measurements and a 2-month landmark analysis both yielded estimates closely matching the primary analysis, which argues against a substantial contribution from this bias. Nevertheless, because the exposure remains a post-baseline variable, the survival results are presented as exploratory and require confirmation in prospective studies with standardized assessment timing. Second, the best response by mRECIST may have been determined before or after the timing of the first AFP measurement, depending on the imaging assessment schedule. Third, post-treatment AFP was not available in 30 patients (15.7%). This missingness reflected institutional monitoring practice rather than early attrition, and the affected patients had less advanced disease and better outcomes; the resulting selection is therefore conservative with respect to the primary association, but reliance on complete-case analysis remains a limitation. Fourth, neither des-gamma-carboxy prothrombin (DCP/PIVKA-II) nor AFP-L3 was measured, because DCP is not reimbursed in Vietnam and AFP-L3 is not available at either participating center; whether combining AFP kinetics with these markers would improve early prediction of response remains an important question for future studies. Fifth, the study has not been validated in an independent population; therefore, the findings, particularly the survival analyses, should be interpreted with caution. Finally, these findings were derived from two oncology centers in Vietnam and may not be directly generalizable to other populations, ethnic groups, or clinical settings; validation in independent, multi-regional cohorts is warranted.

Despite these limitations, the study has several strengths, including a real-world population consecutively enrolled at two centers, mRECIST-based response assessment, the flexible AFP-timing strategy discussed above (which retains more patients than a single fixed time point design), multiple pre-specified AFP response thresholds, adjustment for assessment timing, and analysis of discordant AFP–imaging cases, all of which together clarify the complementary role of AFP in the early assessment of treatment efficacy.

Overall, these findings are consistent with a role for AFP as a complementary biomarker in the early monitoring of lenvatinib response in patients with HCC and pretreatment AFP ≥ 20 ng/mL. A ≥40% AFP decline after treatment initiation was associated with a higher likelihood of imaging response and, in exploratory analyses, with more favorable survival. AFP does not replace mRECIST-based assessment, and any clinical application would need to be established prospectively; for now, AFP results should be interpreted alongside imaging, clinical course, and liver function.

## 5. Conclusions

In this retrospective two-center cohort of patients with HCC treated with lenvatinib who had pretreatment AFP ≥ 20 ng/mL, a ≥40% AFP decline at the first post-treatment measurement was independently associated with ORR and DCR according to mRECIST, and this association persisted in sensitivity and landmark analyses. Patients with pretreatment AFP < 20 ng/mL had more favorable survival outcomes but were not suitable for evaluation using the ≥40% AFP decline criterion. Using the first available AFP result reflects real-world practice and retains more patients than requiring testing at a single fixed time point. These findings are hypothesis-generating. Given the retrospective design, the modest sample size, the two-center setting, and the absence of external validation, AFP kinetics cannot yet be recommended for routine clinical decision-making; confirmation in prospective, multicenter studies with an independent validation cohort is required.

## Figures and Tables

**Table 1:** Baseline characteristics of the study population.

Characteristic	Value (*N* = 191)
Age, years, median (IQR)	60.0 (54.0–65.0)
Male sex, *n* (%)	172 (90.1%)
Hepatitis B virus infection, *n* (%)	97 (50.8%)
Hepatitis C virus infection, *n* (%)	6 (3.1%)
Diabetes mellitus, *n* (%)	25 (13.1%)
Alcohol use, *n* (%)	41 (21.5%)
ECOG PS 0/1/≥2, *n* (%)	142 (74.3)/33 (17.3)/16 (8.4)
Child–Pugh score A5/A6, *n* (%)	153 (80.1)/38 (19.9)
ALBI grade 1/2/3, *n* (%) *	88 (46.1)/92 (48.2)/2 (1.0)
BCLC stage B/C, *n* (%)	50 (26.2)/141 (73.8)
Macrovascular invasion, *n* (%)	85 (44.5%)
Portal vein tumor thrombus, VP4, *n* (%)	40 (20.9%)
Extrahepatic metastasis, *n* (%)	87 (45.5%)
Tumor size, cm, median (IQR)	7.5 (4.4–10.4)
Intrahepatic tumor number, median (IQR)	2.0 (1.0–5.0)
Intrahepatic tumor number ≥ 5, *n* (%) **	70/183 (38.3%)
Pretreatment AFP, ng/mL, median (IQR)	354.6 (20.1–1371.2)
Pretreatment AFP ≥ 20 ng/mL, *n* (%) ‡	143/190 (75.3%)
Pretreatment AFP ≥ 400 ng/mL, *n* (%) ‡	92/190 (48.4%)
Prior local treatment (surgery/TACE/RFA), *n* (%)	88 (46.1%)
of which: surgery/TACE/RFA, *n* ¶	28/72/8
Lenvatinib treatment duration, months, median (IQR)	5.0 (3.0–7.0)
Reason for stopping lenvatinib ‖: disease progression/toxicity/other, *n* (%)	117 (70.5)/5 (3.0)/44 (26.5)

IQR: interquartile range. Percentages refer to the overall population (N = 191) unless otherwise noted. * ALBI grade was missing for 9 patients (4.7%). ** Tumor number was missing for 8 patients (4.2%); percentage calculated among the 183 patients with available data. ‡ Pretreatment AFP was missing for 1 patient (0.5%); percentage calculated among the 190 patients with available data. ‖ The reason for discontinuation was documented for 166 patients; the remaining 25 comprised 23 who were still on treatment and 2 who had discontinued for an unspecified reason at the time of data lock. ¶ Some patients received more than one modality; the sum of modalities therefore exceeds the number of patients who received prior local treatment. ECOG: Eastern Cooperative Oncology Group; ALBI: albumin–bilirubin; BCLC: Barcelona Clinic Liver Cancer; VP4: tumor thrombus in the main portal trunk (Vp4 of the Liver Cancer Study Group of Japan classification); TACE: transarterial chemoembolization; RFA: radiofrequency ablation; AFP: alpha-fetoprotein.

**Table 2:** Timing of the first post-treatment AFP measurement in the primary analysis population.

Timing of First AFP	No. of Patients	Percentage (%)
Cycle 1	99	79.8
Cycle 2	19	15.3
Cycle 3	4	3.2
Cycle 4	2	1.6
Total	124	100

One treatment cycle was defined as 30 days; therefore, cycles 1, 2, 3, and 4 correspond to approximately 1, 2, 3, and 4 months after lenvatinib initiation, respectively.

**Table 3:** Response according to AFP decline of ≥40% status.

Outcome	AFP Response (*n* = 47)	No AFP Response (*n* = 77)	OR (95% CI)	*p*
ORR	21 (44.7%)	5 (6.5%)	11.36 (3.68–42.67)	<0.001
DCR	39 (83.0%)	44 (57.1%)	3.62 (1.42–10.19)	0.003

**Table 4:** Multivariable logistic regression models.

Variable	OR (ORR)	95% CI (ORR)	*p* (ORR)	OR (DCR)	95% CI (DCR)	*p* (DCR)
AFP response ≥40% (primary exposure)	12.96	3.85–43.66	<0.001	5.76	1.95–17.00	0.002
Timing of first AFP after cycle 1	1.75	0.48–6.31	0.394	0.48	0.16–1.48	0.202
Pretreatment ALBI score	0.83	0.23–3.01	0.783	0.74	0.27–2.02	0.553
Macrovascular invasion	1.43	0.46–4.43	0.537	0.95	0.40–2.25	0.903
Tumor number ≥ 5	0.80	0.26–2.49	0.702	0.50	0.21–1.22	0.129

Models adjusted for timing of first AFP after cycle 1, pretreatment ALBI, macrovascular invasion, and tumor number ≥ 5 (n = 113, complete-case analysis).

**Table 5:** Analysis of AFP decline thresholds.

Decline Threshold	No. with AFP Response	ORR in Responders	ORR in Non-Responders	OR (95% CI)	*p*
≥20%	60	36.7%	6.3%	8.54 (2.62–36.72)	<0.001
≥30%	54	38.9%	7.1%	8.12 (2.67–30.11)	<0.001
≥40%	47	44.7%	6.5%	11.36 (3.68–42.67)	<0.001
≥50%	43	46.5%	7.4%	10.61 (3.59–36.36)	<0.001

**Table 7:** Treatment outcomes according to pretreatment AFP.

Outcome	Pretreatment AFP < 20 ng/mL (n = 47)	Pretreatment AFP ≥ 20 ng/mL (n = 143)	*p*
ORR, n (%)	17 (36.2%)	31 (21.7%)	0.055
DCR, n (%)	41 (87.2%)	99 (69.2%)	0.021
Median PFS, months	10.58	6.50	0.011 *
Median OS, months	22.37	12.61	0.017 *

ORR and DCR were analyzed using Fisher’s exact test. PFS and OS: Kaplan–Meier estimates; * *p* from the log-rank test.

**Table 8:** Association between AFP response and treatment outcomes stratified by HBV status.

HBV Status	Outcome	AFP Response	No AFP Response	OR/HR (95% CI)	*p*
HBV+ (n = 60)	ORR	45.8% (11/24)	5.6% (2/36)	OR 13.67 (2.50–143.40)	<0.001
	DCR	79.2% (19/24)	58.3% (21/36)	OR 2.67 (0.74–11.25)	0.161
	Median PFS, months	6.60	4.37	HR 0.48 (0.27–0.88)	0.017
	Median OS, months	15.51	8.84	HR 0.58 (0.30–1.10)	0.094
HBV− (n = 64)	ORR	43.5% (10/23)	7.3% (3/41)	OR 9.34 (2.01–60.95)	0.001
	DCR	87.0% (20/23)	56.1% (23/41)	OR 5.09 (1.22–30.93)	0.014
	Median PFS, months	9.23	4.17	HR 0.36 (0.20–0.67)	0.001
	Median OS, months	20.70	11.20	HR 0.35 (0.18–0.71)	0.004

OR (ORR, DCR): Fisher’s exact test, unadjusted. HR (PFS, OS): univariable Cox model (AFP response), unadjusted given the limited sample size of each subgroup; median PFS/OS was estimated by the Kaplan–Meier method. A subgroup analysis by HCV status was not performed because of the very small sample size (n = 4).

**Table 9:** Sensitivity analysis restricted to patients with a first AFP measurement at cycle 1 (n = 99).

Outcome	AFP Response (n = 35)	No AFP Response (n = 64)	Effect Estimate (95% CI)	*p*
ORR, n (%)	13 (37.1)	4 (6.2)	OR 8.64 (2.35–40.32)	<0.001
ORR, adjusted *	—	—	aOR 12.27 (3.01–50.09)	<0.001
DCR, n (%)	28 (80.0)	38 (59.4)	OR 2.71 (0.97–8.48)	0.046
DCR, adjusted *	—	—	aOR 4.48 (1.35–14.85)	0.014
Median PFS, months	8.71	4.30	—	<0.001 †
Median OS, months	18.96	10.15	—	0.002 †

ORR: objective response rate; DCR: disease control rate; PFS: progression-free survival; OS: overall survival; OR: odds ratio; aOR: adjusted odds ratio; CI: confidence interval. Odds ratios are conditional maximum-likelihood estimates from Fisher’s exact test. * Adjusted for pretreatment ALBI score, macrovascular invasion, and tumor number ≥ 5 (n = 92, complete-case analysis). † Log-rank test.

**Table 10:** Landmark analyses of progression-free survival and overall survival according to AFP response.

Landmark	Outcome	No. Analyzed (Events)	Unadjusted HR (95% CI)	*p*	Adjusted HR (95% CI) *	*p*
2 months	PFS	114 (96)	0.38 (0.24–0.59)	<0.001	0.35 (0.21–0.57)	<0.001
2 months	OS	116 (79)	0.45 (0.28–0.73)	0.001	0.48 (0.29–0.79)	0.004
3 months	PFS	92 (74)	0.41 (0.25–0.68)	<0.001	0.42 (0.25–0.72)	0.001
3 months	OS	113 (75)	0.43 (0.26–0.71)	0.001	0.45 (0.27–0.76)	0.003

HR: hazard ratio; CI: confidence interval; PFS: progression-free survival; OS: overall survival. Survival time was recomputed from the landmark. Patients who experienced the event before the landmark were excluded. * Adjusted for pretreatment ALBI score, macrovascular invasion, and tumor number ≥ 5, fitted on complete cases (2-month landmark: n = 104 for PFS, n = 106 for OS; 3-month landmark: n = 84 for PFS, n = 104 for OS).

**Table 11:** Summary of comparisons with prior studies on the association between AFP response and treatment outcomes.

Study	Population/Design	AFP Response Threshold	ORR: Responders vs. Non-Responders	Other Effect Estimate (95% CI)
This study (2026)	HCC, lenvatinib, real-world clinical practice (n = 124)	≥40% decline at first available test	44.7% vs. 6.5%	Adjusted OR for ORR 12.96 (3.85–43.66); HR PFS 0.38; HR OS 0.49
Kodama et al., 2019 [7]	HCC, lenvatinib	Sustained decline, weeks 2–4	67% vs. 0%	*p* = 0.02
Saeki et al., 2020 [8]	HCC, lenvatinib, baseline AFP ≥ 10 ng/mL	≥40% decline at 1 month	68.4% vs. 7.1%	OR 51.39 (4.89–540.28)
Liu et al., 2022 [9]	HBV-related HCC, lenvatinib, baseline AFP ≥ 20 ng/mL	>20% decline at 4 weeks	34.5% vs. 6.3%	Median PFS 13 vs. 7 months
Tian et al., 2023 [10]	Meta-analysis, 3056 patients, targeted/immunotherapy	Varied by original study	—	Pooled HR OS 0.48 (0.40–0.56); pooled HR PFS 0.39 (0.33–0.46)
Zheng et al., 2025 [11]	Multicenter, lenvatinib	4 AFP kinetic patterns	—	Adjusted HR death 0.28 (0.18–0.42); adjusted HR PFS/death 0.34 (0.24–0.47)
Mahipal et al. (REFLECT post hoc), 2026 [12]	REFLECT trial, lenvatinib	≥20% decline at week 8	48.0% vs. 13.7%	Median PFS 7.4 vs. 3.5 months; median OS 13.4 vs. 8.3 months
Tian et al. (Frontiers 2025) [18]	Meta-analysis, 131 studies, immune checkpoint inhibitors	Varied (>20% most common)	—	Pooled OR ORR 5.39 (3.96–7.32); pooled HR OS 0.41 (0.33–0.52); pooled HR PFS 0.38 (0.30–0.47)

Data cited from the original studies [7,8,9,10,11,12,18]; CI: confidence interval. Cross-study comparisons are illustrative only, given differences in study population, AFP response threshold, assessment timing, and analytic method.

## Data Availability

The data supporting the findings of this study can be provided by the corresponding author (Ngoc-Tan Hoang) upon reasonable request and subject to approval by the data management authorities of Vietnam National Cancer Hospital/Hanoi Oncology Hospital; the data are not publicly available because they contain clinical information that could identify patients. The dataset analyzed in this study shares the same base population (191 patients with HCC treated with first-line lenvatinib at Vietnam National Cancer Hospital and Hanoi Oncology Hospital, March 2021–December 2025) as other companion studies from the same research group, including a comprehensive study of survival prognostic factors in the overall population, a study on lenvatinib outcomes within and outside REFLECT eligibility criteria, and a study on inflammatory biomarkers in the same population, currently under review at other journals. These reports address independent, non-overlapping research questions (post-treatment AFP kinetics and mRECIST response in this study; survival prognostic factors in the overall population; stratification by REFLECT criteria; the role of inflammatory biomarkers), cross-reference one another, and the population overlap has been transparently disclosed to the editorial board of each journal.

## Supplementary Materials

The following supporting information can be downloaded at: https://www.mdpi.com/article/10.3390/medicina62091774/s1, Figure S1. Sensitivity analysis of AFP decline threshold for objective response rate (ORR). Forest plot showing odds ratios (OR) and 95% confidence intervals for ORR at four AFP decline thresholds (≥20%, ≥30%, ≥40%, ≥50%) in the primary analysis population (n = 124), corresponding to Table 5 in the main manuscript. The ≥40% threshold (filled dark-blue square) is the pre-specified (a priori) primary analysis threshold; the remaining thresholds (20%, 30%, 50%) are sensitivity analyses. The dashed line indicates OR = 1 (no difference). These results come from a SINGLE cohort study evaluated at different thresholds, not a pooled analysis of multiple studies. Figure S2. Receiver operating characteristic (ROC) curve for continuous AFP change (percentage decline from baseline) predicting objective response by mRECIST in the primary analysis population (n = 124). AUC = 0.794 (95% CI 0.685–0.903). The marked point is the data-derived Youden-optimal cutoff (AFP decline ~53%; sensitivity 76.9%, specificity 79.6%), shown for reference only; it is distinct from the pre-specified ≥40% threshold used in the primary analysis (Table 5). Figure S3. Kaplan–Meier curves for progression-free survival (A) and overall survival (B) measured from the 2-month landmark, stratified by AFP decline ≥40% at the first post-treatment measurement. The landmark corresponds to the end of treatment cycle 2, by which time AFP response status had been determined in 118 of 124 patients (95.2%). Patients who experienced the event before the landmark were excluded (2 for overall survival, 4 for progression-free survival); survival time was recomputed from the landmark. Number-at-risk tables are shown at 6-month intervals. *p*-values are from the log-rank test. Corresponding hazard ratios are reported in Table 10 of the main manuscript. Table S1. Descriptive comparison between patients with and without post-treatment AFP, overall and among patients with pretreatment AFP ≥20 ng/mL. Table S2. STROBE checklist for retrospective cohort observational studies.
