# Supplementary material for: Predictive Value of On-Treatment Alpha-Fetoprotein Change for Lenvatinib Response in Patients with Hepatocellular Carcinoma: A Real-World Retrospective Study in Vietnam
Source: Medicina (Kaunas). 2026 Sep 16;62(9):1774. doi: 10.3390/medicina62091774 (PMC13609512; doi:10.3390/medicina62091774)
Supplement: Supplementary file 1 [file medicina-62-01774-s001.zip › medicina-4542332-supplementary.pdf]

Supplementary Materials

Predictive Value of On-Treatment Alpha-Fetoprotein Change for Lenvatinib Response in Patients with Hepatocellular Carcinoma: A Real-World Retrospective Study in Vietnam

This file accompanies the main manuscript, submitted separately in accordance with the "Supplementary Materials" requirements of Medicina (MDPI).

Figure S1

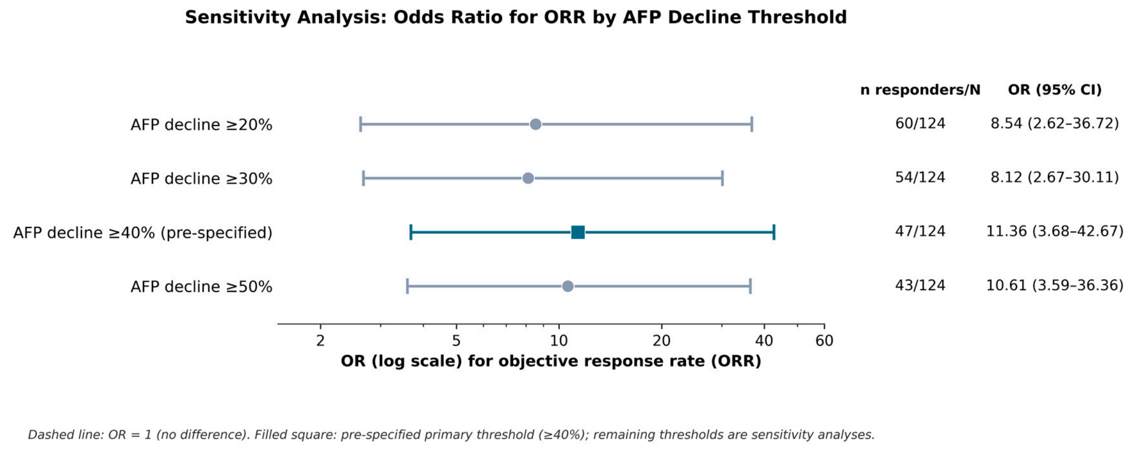

Figure S1. Sensitivity analysis of AFP decline threshold for objective response rate (ORR). Forest plot showing odds ratios (OR) and 95% confidence intervals for ORR at four AFP decline thresholds (≥20%, ≥30%, ≥40%, ≥50%) in the primary analysis population (n=124), corresponding to Table 5 in the main manuscript. The ≥40% threshold (filled dark-blue square) is the pre-specified (a priori) primary analysis threshold; the remaining thresholds (20%, 30%, 50%) are sensitivity analyses. The dashed line indicates OR = 1 (no difference). These results come from a SINGLE cohort study evaluated at different thresholds, not a pooled analysis of multiple studies.

Figure S2

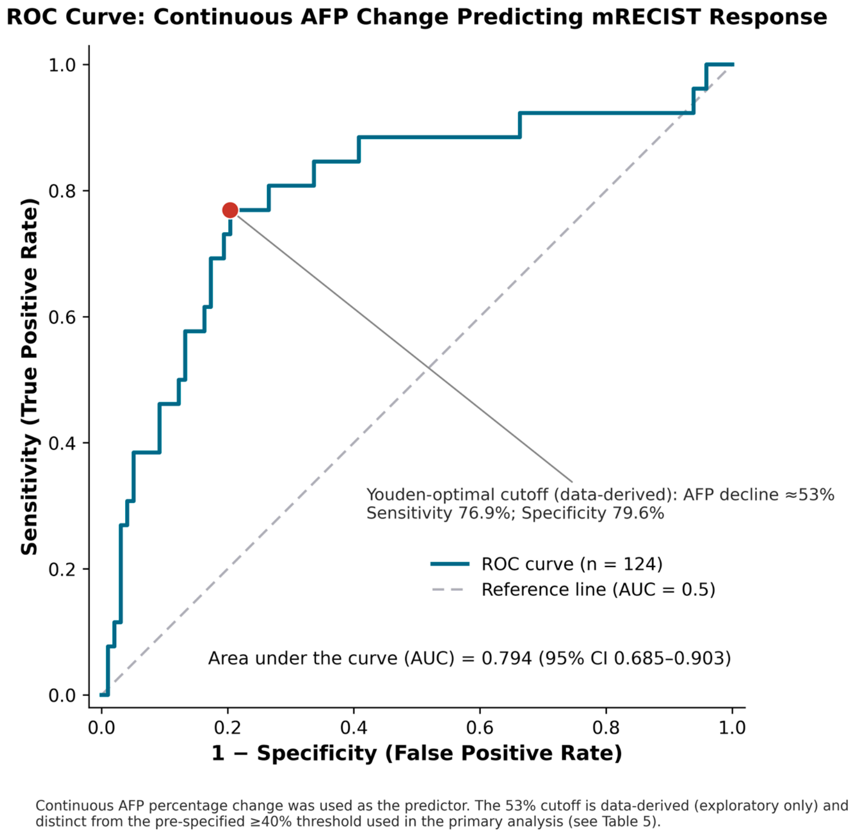

Figure S2. Receiver operating characteristic (ROC) curve for continuous AFP change (percentage decline from baseline) predicting objective response by mRECIST in the primary analysis population (n=124). AUC = 0.794 (95% CI 0.685-0.903). The marked point is the data-derived

Youden-optimal cutoff (AFP decline ~53%; sensitivity 76.9%, specificity 79.6%), shown for reference only; it is distinct from the pre-specified >=40% threshold used in the primary analysis (Table 5).

Figure S3

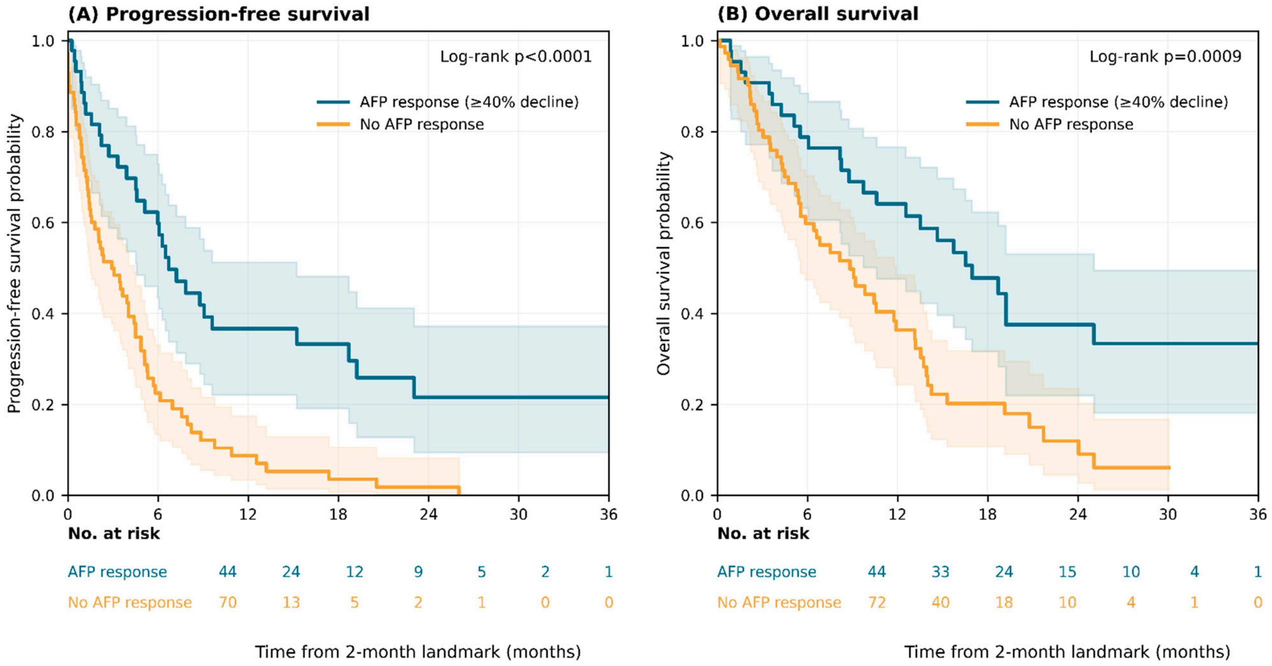

Figure S3. Kaplan–Meier curves for progression-free survival (A) and overall survival (B) measured from the 2-month landmark, stratified by AFP decline ≥40% at the first post-treatment measurement. The landmark corresponds to the end of treatment cycle 2, by which time AFP response status had been determined in 118 of 124 patients (95.2%). Patients who experienced the event before the landmark were excluded (2 for overall survival, 4 for progression-free survival); survival time was recomputed from the landmark. Number-at-risk tables are shown at 6-month intervals. P-values are from the log-rank test. Corresponding hazard ratios are reported in Table 10 of the main manuscript.

Table S1

Descriptive comparison between patients with and without post-treatment AFP, overall and among patients with pretreatment AFP ≥20 ng/mL

Characteristic	Without post-treatment AFP (n = 30)	With post-treatment AFP (n = 161)	p
**Panel A. All patients**			
Age, median (years)	61	60	—
Male, %	90.0	90.1	—
BCLC stage C, %	50.0	78.3	—
Macrovascular invasion, %	23.3	48.4	—
Extrahepatic metastasis, %	36.7	47.2	—
ORR by mRECIST, %	36.7	23.0	—
Median PFS, months	10.86	6.04	—
Median OS, months	11.76	10.84	—
**Panel B. Patients with pretreatment AFP ≥20 ng/mL***	**Without post-treatment AFP (n = 19)**	**Primary analysis population (n = 124)**	**p**

Lenvatinib duration, median (months)	6.5	4.0	0.015
BCLC stage C, %	57.9	79.8	0.044
Macrovascular invasion, %	31.6	53.2	0.090
Extrahepatic metastasis, %	42.1	45.2	1.000
Pretreatment AFP, median (ng/mL)	532.8	1210.0	0.255
ORR by mRECIST, %	26.3	21.0	0.562
DCR by mRECIST, %	84.2	66.9	0.183
Death, %	36.8	67.7	0.019
Progression or death, %	52.6	84.7	0.003
Death within the first 3 months, n (%)	0 (0.0)	7 (5.6)	—
Median PFS, months	12.29	5.95	0.014
Median OS, months	Not reached	12.58	0.084

Panel A is descriptive only; no formal statistical testing was performed. *Panel B compares the 19 patients with pretreatment AFP ≥20 ng/mL who lacked a post-treatment AFP with the 124 patients of the primary analysis population; p-values are from Fisher's exact test (proportions), the Mann–Whitney U test (medians of continuous variables), or the log-rank test (PFS and OS). None of the 30 patients without a post-treatment AFP had an AFP result recorded at any of the 12 follow-up cycles, and none died within the first 3 months; the proportion without post-treatment AFP differed between the two participating hospitals (9/107, 8.4% vs. 21/84, 25.0%; p=0.002). See interpretation in Section 3.1 and the Limitations subsection of the main manuscript. Extrahepatic metastasis was defined consistently to include abdominal lymph node metastasis, per the final dataset shared across the companion studies.

Table S2

STROBE checklist for retrospective cohort observational studies

Cross-check of the manuscript against the STROBE Statement — checklist of items for cohort studies (von Elm E, et al. STROBE Statement, www.strobe-statement.org). The "Status" column was self-assessed by the author team; all items now meet "Complete" status.

Item	Recommendation (STROBE – cohort study)	Location in manuscript	Status
1a	Indicate the study's design with a commonly used term in the title or the abstract.	Title; Abstract	Complete
1b	Provide in the abstract an informative and balanced summary of what was done and what was found.	ABSTRACT (structured)	Complete
2	Explain the scientific background and rationale for the investigation being reported.	Section 1, paragraphs 1–3	Complete
3	State specific objectives, including any prespecified hypotheses.	Section 1, final paragraph	Complete
4	Present key elements of study design early in the paper.	Section 2.1	Complete

5	Describe the setting, locations, and relevant dates, including periods of recruitment, exposure, follow-up, and data collection.	Section 2.1 (setting; recruitment period March 2021–December 2025)	Complete
6a	Give the eligibility criteria, and the sources and methods of selection of participants.	Section 2.1; Figure 1	Complete
6b	Matching criteria (for matched studies).	Not applicable	Not applicable
7	Clearly define all outcomes, exposures, predictors, potential confounders, and effect modifiers.	Sections 2.2–2.4	Complete
8	For each variable of interest, give sources of data and details of methods of assessment.	Sections 2.2–2.3	Complete
9	Describe any efforts to address potential sources of bias.	Section 2.1 (Table S1); Sections 2.5 and 3.10 (landmark analysis for immortal-time bias); Discussion (Limitations)	Complete
10	Explain how the study size was arrived at.	Section 2.1	Complete
11	Explain how quantitative variables were handled; describe grouping if applicable.	Section 2.3 (40% AFP threshold; sensitivity analysis at 20/30/50%)	Complete
12a	Describe all statistical methods, including those used to control for confounding.	Section 2.5	Complete
12b	Describe methods used to examine subgroups and interactions.	Sections 2.5; 3.8	Complete
12c	Explain how missing data were addressed.	Section 2.1 (pretreatment AFP = 0 treated as missing); Section 2.5 (complete-case analysis); Section 3.1 and Table S1 (patients without post-treatment AFP)	Complete
12d	Describe how loss to follow-up was addressed.	Figure 1; Table S1	Complete
12e	Sensitivity analyses.	Section 3.4 and Table 5 (alternative AFP thresholds); Section 3.9 and Table 9 (cycle-1 sensitivity analysis); Section 3.10 and Table 10 (landmark analysis)	Complete
13a	Report the numbers of individuals at each stage of the study.	Figure 1	Complete
13b	Give reasons for non-participation at each stage.	Figure 1; Section 3.1; Table S1	Complete
13c	Consider use of a flow diagram.	Figure 1	Complete

14a	Give characteristics of study participants and information on exposures and potential confounders.	Table 1	Complete
14b	Indicate the number of participants with missing data for each variable of interest.	Table 1 (footnote)	Complete
14c	Summarize follow-up time (e.g., average and total amount).	Section 2.5; 3.1	Complete
15	Report numbers of outcome events or summary measures over time.	Tables 3, 4, 7, 9, 10	Complete
16a	Give unadjusted and adjusted estimates and their precision; specify confounders adjusted for.	Tables 3, 4, 7, 9, 10	Complete
16b	Report category boundaries when continuous variables were categorized.	Table 5	Complete
16c	Translate relative risk into absolute risk for a meaningful time period, if relevant.	Section 3.2 (absolute ORR 44.7% vs. 6.5%)	Complete
17	Report other analyses done (subgroups, interactions, sensitivity analyses).	Sections 3.4, 3.5, 3.7, 3.8, 3.9, 3.10	Complete
18	Summarize key results with reference to study objectives.	Section 4, paragraph 1	Complete
19	Discuss limitations, sources of potential bias or imprecision, and direction and magnitude of any potential bias.	Section 4 (Limitations)	Complete
20	Give a cautious overall interpretation considering objectives, limitations, multiplicity of analyses, and relevant evidence.	Section 4 (throughout)	Complete
21	Discuss the generalizability (external validity) of the study results.	Section 4 (Limitations)	Complete
22	Give the source of funding and the role of the funders.	FUNDING	Complete
